# From farm to table: assessing the status and health risk assessment of heavy metal pollution in rice in Henan Province

**DOI:** 10.3389/fpubh.2025.1499653

**Published:** 2025-04-17

**Authors:** Yumeng Yan, Zhenxing Mao, Xinlu Wang, Zhiwei Chen, Cuicui Ma, Dandan Wei, Wenjing Yan, Xueyan Wu, Yao Guo, Haoran Xu, Guozhen Han, Erbao Han, Huilin Lou, Taimeng Chen, Wenqian Huo, Chongjian Wang, Shan Huang, Xin Zeng

**Affiliations:** ^1^Department of Epidemiology and Biostatistics, College of Public Health, Zhengzhou University, Zhengzhou, China; ^2^Collaborative Innovation Center of Prevention and Treatment of Major Diseases by Chinese and Western Medicine, Zhengzhou, China; ^3^Department of Occupational and Environmental Health Sciences, College of Public Health, Zhengzhou University, Zhengzhou, China; ^4^Institute for Special Food Inspection, Henan Province Food Inspection Research Institute, Zhengzhou, China; ^5^Department of Social Medicine, College of Public Health, Zhengzhou University, Zhengzhou, China

**Keywords:** heavy metal, rice safety, health risk assessment, environmental pollution, agricultural impact

## Abstract

**Introduction:**

In recent years, industrial and agricultural advancements in Henan Province have increased heavy metal contamination in rice, raising public concerns. The aim of this study was to investigate the contamination levels of five heavy metals, cadmium (Cd), chromium (Cr), lead (Pb), mercury (Hg), and inorganic arsenic (As), in rice from Henan Province and to assess potential health risks.

**Methods:**

A total of 6,632 rice samples were collected from 18 regions between 2020 and 2022. The samples were analyzed for Cd, Cr, Pb, Hg, and As using inductively coupled plasma mass spectrometry (ICP-MS). Detection rates were compared using the chi-square test, and health risks were assessed for the three populations (adults, children and toddlers) according to USEPA guidelines.

**Results:**

The results of the study showed that As had the highest detection rate of 99.59%, followed by Cd (27.69%), Cr (22.57%), Pb (2.25%) and Hg (1.95%). Cd levels were significantly higher in urban areas (30.42%) than rural areas (23.13%) (*P* < 0.001). The detection rate of Cd was higher in the southern region compared to other regions, while the detection rate of Cr in the eastern region was the highest among all regions. The health risk assessment showed that the Hazard Quotient (HQ) for inorganic As exceeded 1. However, children and toddlers were at relatively higher risk of exposure to As compared to adults.

**Conclusion:**

This study revealed the current status of heavy metal contamination in rice in Henan, particularly highlighting the presence of As as a significant health hazard for children and toddlers. Consequently, it is recommended that the relevant authorities should strengthen the monitoring and control of food safety in order to effectively protect public health.

## 1 Introduction

Rapid economic and social development in China, along with population growth, urbanization, industrialization, and the expansion of heavy metal-related industries, has led to significant heavy metal accumulation in agricultural soils, posing a serious threat to crop quality, safety, and human health (1). Heavy metals such as cadmium (Cd), chromium (Cr), lead (Pb), total mercury (Hg) and inorganic arsenic (As), are often collectively referred to as the “five poisonous elements” because of their toxicity and potential health hazards. These heavy metals primarily enter the human body through the diet and accumulate via the food chain, causing health issues (2). Cadmium mainly accumulates in the kidneys, which may lead to renal insufficiency and osteoporosis (3). Chromium is a heavy metal needed by the human body, but excessive intake can cause poisoning, damage the liver and kidneys, and increase the risk of cancer (4). Lead can damage the digestive system, liver, kidneys, and nervous system, causing high blood pressure, infertility, and affecting the intellectual development of children (5). Long-term exposure to mercury is associated with Minamata disease and neonatal neurological problems in newborns (6). Arsenic, though a metalloid, is classified as a heavy metal due to its toxicity. Excessive exposure can cause cardiovascular disorders and cancer (7).

The United Nations Food and Agriculture Organization reports that the worldwide rise in population has increased the demand for rice, with a particularly notable surge in China. As a key food crop, rice can absorb and accumulate significant amounts of heavy metals, potentially posing health risks through rice-based products (8). In regions where rice constitutes the primary food staple, notably Asia and Latin America, the consumption rate of rice and its derivative products is exceptionally high, thereby rendering food safety concerns in these areas particularly acute (9). Several cross-sectional surveys have examined the levels of heavy metals in rice from different countries. For example, studies in India showed geometric mean concentrations (mg/kg) of As, Cd, Cr, and Pb in rice samples were 0.065, 0.008, 0.281, and 0.792, respectively, which exceeded the limits of the Indian Food Standards (10). The mean levels of the elements in the Vietnamese rice samples followed a descending order of Cr (0.296 mg/kg) > As (0.115 mg/kg) > Cd (0.111 mg/kg) > Pb (0.075 mg/kg) > Hg (0.007 mg/kg) (11). Numerous studies have evaluated the potential risk associated with various heavy metals present in collected rice samples, and their findings suggest that the consumption of rice contaminated with metals like As, Cd and Pb poses a considerable peril to human health. For example, exposure of Bangladeshis to As through rice consumption may result in non-carcinogenic and carcinogenic health risks (12, 13). A study involving children, adolescents, and adults in the United States has unveiled a correlation between rice intake and elevated levels of arsenic in the human body (14).

China, as the country with the largest population in the world, is gradually increasing the proportion of refined grains, especially rice, in its staple food (15). However, alongside its rapid economic development, China has experienced serious soil heavy metal pollution (16). By 2022, the area under rice cultivation in China covered 29,921.2 thousand hectares. It has been reported that the exceeding rate of metal elements in Chinese arable soils is as high as 1.71%, and the proportion of heavy metal contamination in soils that need to be fallowed is 15.58%, and this situation is particularly serious in Henan and Hunan (17). Some studies have examined the heavy metal content in rice. A comprehensive analysis measured the concentrations of 10 heavy metals in rice in Bay County Xinjiang, China, finding that As posed the highest risk of causing cancer during consumption (18). Additionally, a study of rice samples covering 17 provinces (cities) in China revealed that all rice samples had a heavy metal hazard index of more than 1, with As having the most significant impact (19). Similarly, the study in Fujian region indicated that the mean concentrations of Cd, Hg, As, Pb, and Cr in rice were 0.064, 0.002, 0.464, 0.072, and 0.138 mg/kg, respectively, and highlighted that Arsenic contributes the most to the health risk of children and adults (20).

As an important grain province and resource reserve base in China, the safety and quality of grain production in Henan Province is directly related to the dietary health of millions of people. As a major grain crop in Henan Province, the quality and security of rice is of great concern. Therefore, investigating the content and spatial distribution of heavy metals in rice and evaluating the health hazards to human beings through dietary intake will not only help us to understand the status of heavy metal pollution in the main grain producing areas and its health impacts, but also provide a reference for the management of heavy metal pollution in other areas.

## 2 Methods and materials

### 2.1 Sampling

**Figure 1:**
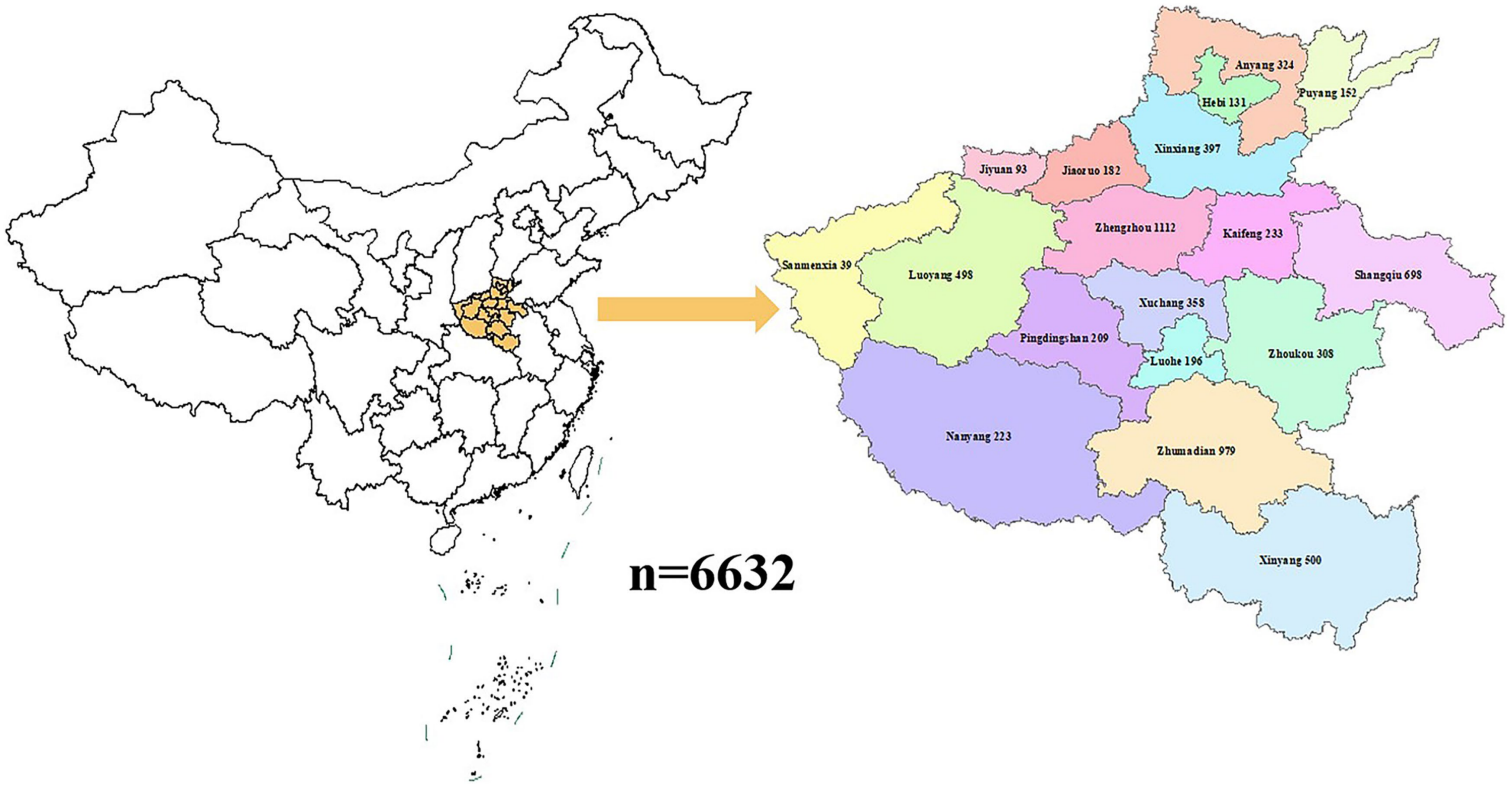
The spatial distribution and the concentrations of heavy metals in the collected samples from various regions in Henan Province.

The location and number of rice samples collected were shown in Figure 1. In this study, 6,632 rice samples from 2020 to 2022 were selected for heavy metals testing. These samples were randomly selected from 18 prefecture-level cities in Henan Province. Among them, 3,912 samples originated from urban areas and the other 2,720 samples were from rural areas. These rice samples were collected from different sales locations such as supermarkets, wholesale markets, shopping centers, and farmers’ markets. During the sampling process, rigorous measures were implemented to ascertain the origin of rice samples exclusively from Henan Province. Initially, rice labeled as grown in Henan Province was inquired about and screened to ensure geographical specificity. Subsequently, information on rice cultivation in Henan Province was researched to enhance representative sampling. Furthermore, sampled unit information was documented to facilitate retrospective review and uphold the integrity and reliability of the data collected. All rice samples were obtained through regular market channels.

### 2.2 Sample preparation and analysis

The elemental contents of Cr, As, Hg, Pb and Cd in rice samples were determined by inductively coupled plasma mass spectrometry (ICP-MS).

The collected rice samples were crushed into powder, and 0.3 g (accurate to 0.001 g) of this powder was weighed into a microwave dissolution cup. Then, 7 mL of nitric acid was added and left for 1 h with the lid on. Afterward, the lid was tightened and the sample was dissolved according to the standard procedure of microwave dissolution (Supplementary Table S1). After cooling, the lid was slowly opened to exhaust, the inner lid was rinsed with a small amount of water, the tank was placed on a temperature-controlled hot plate or an ultrasonic water bath and heated at 100°C for 30 min, and then, water was added to bring the volume up to 25 mL. The mixture was stirred well and reserved. For quality control, double-parallel samples, blank samples spiked with recovery and quality control samples were used for quality control to ensure the accuracy of the experimental data, and retesting tests were carried out for the exceeding samples.

### 2.3 Daily intake estimation and risk assessment

The cumulative risk to humans from heavy metals in rice was assessed using the health risk assessment methodology for human exposure to contaminants developed by the United States Environmental Protection Agency. The Estimated daily intake (EDI) of heavy metals was calculated according to Equation 1, and the hazard quotient (HQ) was used as the model for health risk assessment according to Equation 2:


(1) $$EDI=\left(C\times IR\right)/BW$$

where EDI (μg/kg bw/day) represents the daily intake of heavy metals through rice. C (μg/kg) is its median concentrations determined in rice, IR (g/day) is the daily ingestion rate of rice, and BW (kg) is body weight. Average IR and BW values for toddlers, children and adults used in these calculations are shown in Supplementary Table S2.


(2) HQ=EDI/RfD

where HQ measures the health risk of heavy metals intake through rice consumption. It assesses the risk by comparing the ratio of the consumption exposure to the safe intake reference dose (RfD) of these elements. When the HQ value exceeds 1, it suggests that the intake of heavy metals may have exceeded the recommended safe levels, which may pose a health risk. Taking the long-term health reference doses of common heavy metals in rice as an example, the RfDs are 0.001 mg/kg/day for Cd, 0.003 mg/kg/day for Cr, 0.004 mg/kg/day for Pb, 0.0001 mg/kg/day for Hg, and 0.0003 mg/kg/day for As.

### 2.4 Statistical analysis

Statistical analysis was performed using IBM SPSS Version 26.0. When the trace element concentration of a sample was less than its LOD, LOD/2 was used instead of the recorded concentration for analysis. *p* < 0.05 was considered statistically significant. In this study, the chi-square test was used to analyze the detection rate of heavy metals in rice between different regions, urban and rural areas. As a non-parametric test, the chi-square test offered a significant advantage in evaluating the variability of the frequency distributions of two or more categorical variables. It was effectively used to identify the differences in the detection rate of heavy metals among regions, thereby revealing the influence of regional factors on the contamination of rice with heavy metals.

## 3 Results

### 3.1 Detection rates and concentrations of heavy metals in rice

The results and detection rates of heavy metals As, Hg, Cr, Pb and Cd in 6,632 test rice samples collected from Henan Province were presented in Table 1. The detection rates of Cd, Cr, Pb, Hg, and As were 27.69, 22.57, 2.25, 1.95, and 99.59%, with median concentrations of 0.002 mg/kg, 0.015 mg/kg, 0.025 mg/kg, 0.002 mg/kg, and 0.110 mg/kg, respectively. The data showed the distribution characteristics of different heavy metals in rice, with As levels particularly elevated. This high prevalence indicated that As element was widely distributed in the soil environment of Henan Province, and there was a risk of rice contamination in Henan Province, while the detection rates of Cd, Cr, Pb and Hg were relatively low. According to GB 2762–2017 National Standard for Food Safety Limits of Contaminants in Food, the limits of Pb, Hg, As, Cd and Cr are 0.2 mg/kg, 0.02 mg/kg, 0.2 mg/kg, 0.2 mg/kg, 1.0 mg/kg, respectively. The 6,632 rice samples examined adhered to these limits, except for Cd.

**Table 1:** Detection rates and concentrations of heavy metals in rice samples (mg/kg).

Heavy metals (*n* = 6,632)	Cd	Cr	Pb	Hg	As
Mean	0.013	0.032	0.026	0.003	0.112
Median	0.002	0.015	0.025	0.002	0.110
SD	0.031	0.049	0.010	0.002	0.030
25th	0.002	0.015	0.025	0.002	0.092
50th	0.002	0.015	0.025	0.002	0.110
75th	0.005	0.015	0.025	0.005	0.130
95th	0.096	0.105	0.025	0.005	0.170
Min	0.002	0.015	0.020	0.002	0.005
Max	0.260	0.560	0.188	0.011	0.190
DR (%)	27.69	22.57	2.25	1.95	99.59
Number of exceeding limit	1	0	0	0	0
ER (%)	0.04	0	0	0	0

DR, detection rate; ER: exceeding rate.

### 3.2 Comparison with the concentrations of heavy metals in rice samples from different countries and regions

An exhaustive comparative analysis of heavy metal contents in samples from Henan Province with those from other Chinese provinces and international samples was presented in Table 2. The results showed that within China, the average content of Cd and Cr in the samples of Henan Province was significantly lower than that of other provinces. Regarding Pb, Henan samples had higher average content than Guizhou, but were lower compared to other provinces. For Hg, the average content in Henan’s samples was found to be higher than that of Heilongjiang, albeit it was still less than the levels recorded in Hunan and Fujian. In terms of As, Henan samples had lower average content than Hunan, but higher than Jiangsu, Heilongjiang, and Fujian. Taken together, the heavy metal contents in Henan Province are characterized by significant geographical features, especially the low contents of Cd and Cr and the relatively high contents of As, which reflect the unique environmental conditions and pollution characteristics of Henan Province. In international comparison, the contents of the five heavy metals in rice in Henan Province were generally lower than those reported in Vietnam and Korea, but the levels of cadmium (Cd) and arsenic (As) were slightly higher than those reported in India.

**Table 2:** Comparison with the concentrations of heavy metals in rice samples from different countries and regions (mg/kg).

Location	Cd	Cr	Pb	Hg	As	Reference
Henan, China	0.013	0.032	0.026	0.003	0.112	This study
Heilongjiang, China	0.0071	0.0755	0.0426	N.D.	0.0990	Li et al. (19)
Jiangsu, China	0.0226	0.0647	0.158	–	0.0802	Li et al. (19)
Hunan, China	–	1.28	0.660	0.069	0.48	Cui et al. (33)
Guizhou, China	0.28	0.215	0.013	–	–	Zhao et al. (34)
Fujian, China	0.076	0.318	0.057	0.01	0.107	Guo et al. (35)
India	0.008	0.281	0.792	–	0.065	Giri and Singh (10)
Viet Nam	0.111	0.296	0.075	0.007	0.115	Chu et al. (11)
Korea	0.04	–	0.111	–	0.237	Lee et al. (36)

N.D.: Not detected; -: The article did not provide relevant data.

### 3.3 Description of the detected concentrations and comparison of detection rates of heavy metals in urban and rural

**Figure 2:**
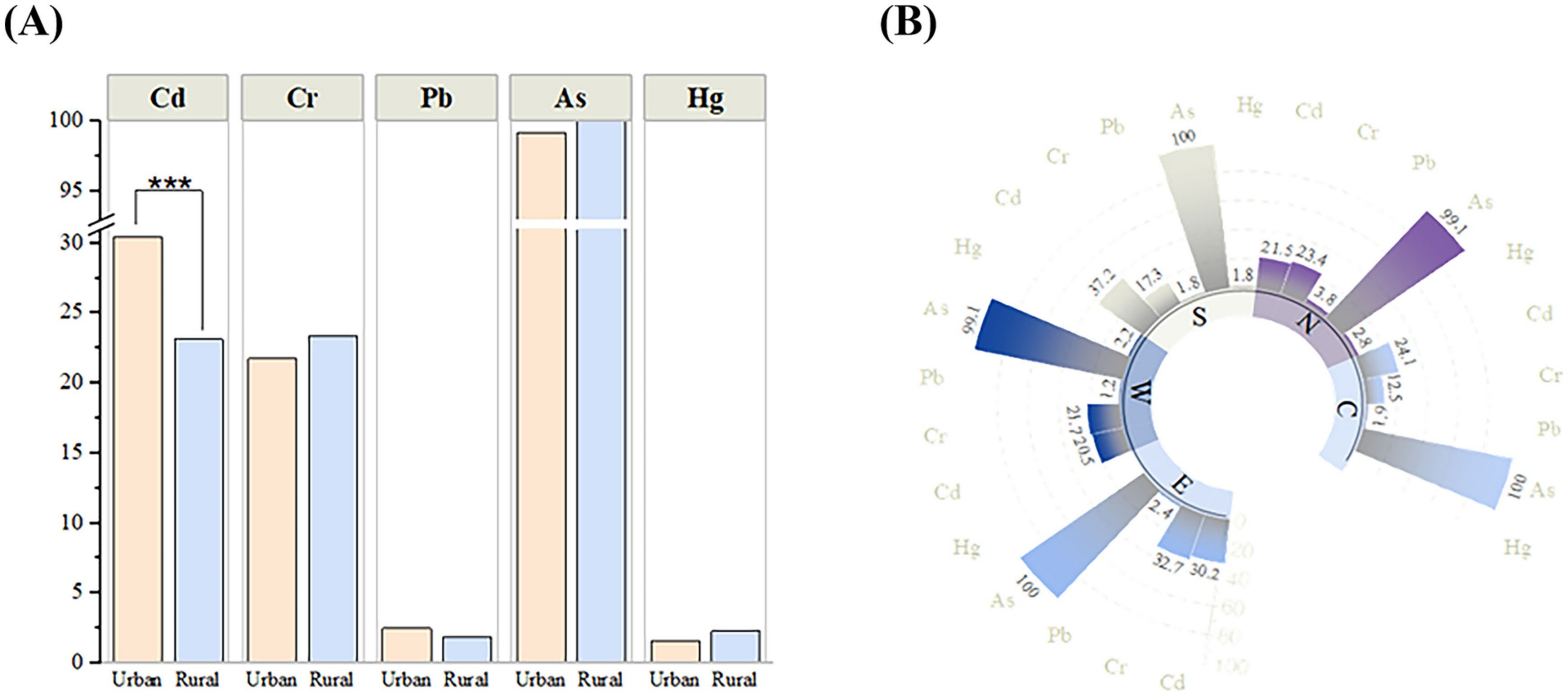
**(A)** Comparison of detection rates of various heavy metals between urban and rural. **(B)** Comparison of the detection rates of various heavy metals in rice samples in different regions of Henan Province. C, Central region of Henan; N, Northern Henan; S, Southern Henan; W, Western Henan; E, Eastern Henan.

After stratifying the rice samples by place of origin (urban and rural), Table 3 revealed the distribution of heavy metal elements in rice from different regions. The analyzed data showed that the average level of Cd in rice in urban areas was 0.015 mg/kg, which was higher than rural areas. On the contrary, the average levels of As and Cr contents in rice in rural areas were higher at 0.112 mg/kg and 0.035 mg/kg, respectively. Figure 2A further compared the detection rate of heavy metals in urban and rural rice, and the specific values were presented in Supplementary Table S3. The Cd detection rate in urban rice was 30.42%, significantly higher than 23.13% in rural rice, and the difference was statistically significant (*p* < 0.001). However, there was no significant difference in the detection rates of Cr, Pb, Hg, and As.

**Table 3:** The levels of heavy metals in rice samples in urban and rural areas of Henan Province (mg/kg).

Site		Cd	Cr	Pb	Hg	As
Urban	Mean	0.015	0.030	0.026	0.003	0.111
	Median	0.002	0.160	0.160	0.002	0.110
	95th	0.110	0.100	0.025	0.005	0.170
	SD	0.034	0.038	0.010	0.002	0.031
	Min	0.002	0.015	0.025	0.002	0.005
	Max	0.026	0.300	0.188	0.011	0.190
Rural	Mean	0.010	0.035	0.026	0.003	0.112
	Median	0.002	0.015	0.025	0.002	0.110
	95th	0.059	0.120	0.025	0.005	0.160
	SD	0.025	0.058	0.009	0.002	0.028
	Min	0.002	0.015	0.020	0.002	0.020
	Max	0.170	0.056	0.157	0.011	0.190

### 3.4 Comparison of the detection rates of various heavy metals in rice samples from different regions of Henan Province

**Figure 3:**
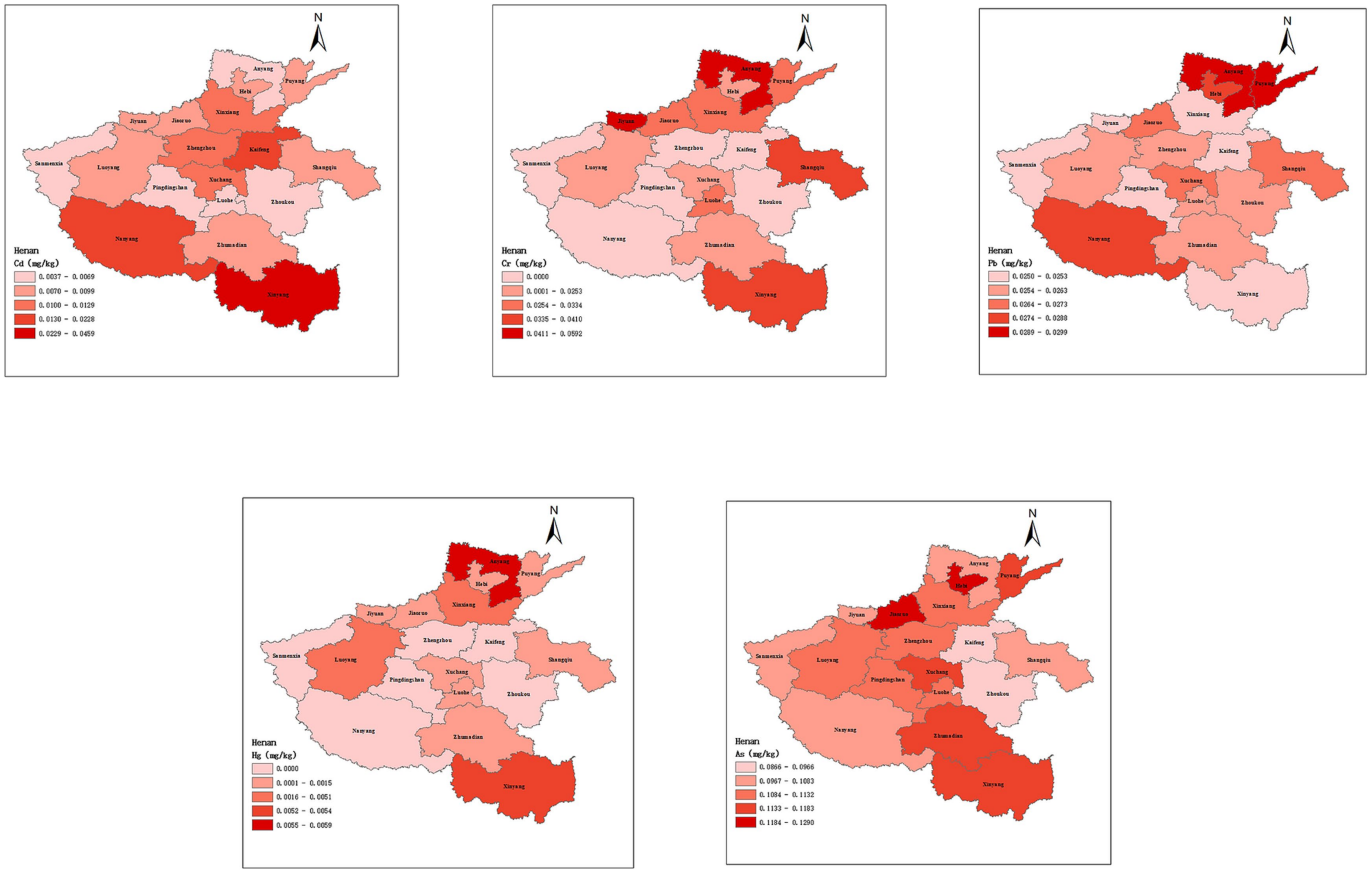
Spatial distribution and concentration of heavy metals in samples collected from 18 districts in Henan Province.

Henan Province was strategically segmented into five distinct regions, namely east, west, south, north, and central, in accordance with its unique topographical features and river networks. The Table 4 presented the heavy metal concentrations measured within each of these regions, offering a distribution of heavy metals detections across the five regions. The average cadmium (Cd) concentration in rice in the southern region reached 0.02 mg/kg, and this level was significantly higher than the Cd concentration in rice in the other four regions. Meanwhile, chromium (Cr) concentration in rice in the eastern region was also characterized as significantly higher than the other regions (the levels of the five heavy metals in rice in 18 cities are shown in Figure 3). After analyzing the data in Figure 2B, it was found that the detection rates of Cd and Cr in rice samples were statistically significant (*p* < 0.05) among different regions (the specific values were presented in Supplementary Table S4). Specifically, the highest Cd detection rate was 37.2% in southern Henan, significantly higher than the 20.5% in the western region. In terms of chromium, the highest detection rate was 32.7% in the eastern part of Henan. In contrast, the detection rates of Pb, As and Hg did not differ significantly between regions, which may indicate that the distribution of these three elements was more uniform or less influenced by regional environmental factors.

**Table 4:** The levels of heavy metals in rice samples from different areas in Henan province (mg/kg).

Site		Cd	Cr	Pb	Hg	As
Eastern Henan (*n* = 1,239)	Mean	0.011	0.041	0.026	0.002	0.104
	Median	0.002	0.015	0.025	0.002	0.100
	95th	0.062	0.170	0.025	0.002	0.160
	SD	0.028	0.061	0.007	0.000	0.028
	Min	0.002	0.015	0.025	0.002	0.048
	Max	0.170	0.400	0.085	0.002	0.180
Western Henan (*n* = 537)	Mean	0.008	0.025	0.026	0.005	0.113
	Median	0.002	0.015	0.025	0.005	0.110
	95th	0.023	0.092	0.025	0.005	0.180
	SD	0.024	0.023	0.005	0.001	0.032
	Min	0.002	0.015	0.025	0.005	0.005
	Max	0.180	0.100	0.075	0.011	0.190
Southern Henan (*n* = 1702)	Mean	0.020	0.030	0.026	0.003	0.116
	Median	0.002	0.015	0.025	0.002	0.110
	95th	0.122	0.111	0.025	0.005	0.170
	SD	0.039	0.043	0.008	0.002	0.028
	Min	0.002	0.015	0.025	0.002	0.045
	Max	0.180	0.400	0.128	0.011	0.190
Northern Henan (*n* = 1,279)	Mean	0.010	0.034	0.027	0.004	0.114
	Median	0.002	0.015	0.025	0.005	0.110
	95th	0.058	0.137	0.025	0.005	0.160
	SD	0.025	0.059	0.013	0.002	0.030
	Min	0.002	0.015	0.025	0.002	0.005
	Max	0.180	0.560	0.157	0.011	0.180
Central region of Henan (*n* = 1875)	Mean	0.011	0.023	0.026	0.002	0.120
	Median	0.002	0.015	0.025	0.002	0.110
	95th	0.077	0.078	0.025	0.002	0.170
	SD	0.030	0.023	0.011	0.000	0.029
	Min	0.002	0.015	0.020	0.002	0.050
	Max	0.260	0.140	0.188	0.002	0.190

### 3.5 Human health risk assessment

#### 3.5.1 EDIs of heavy metals via rice

**Figure 4:**
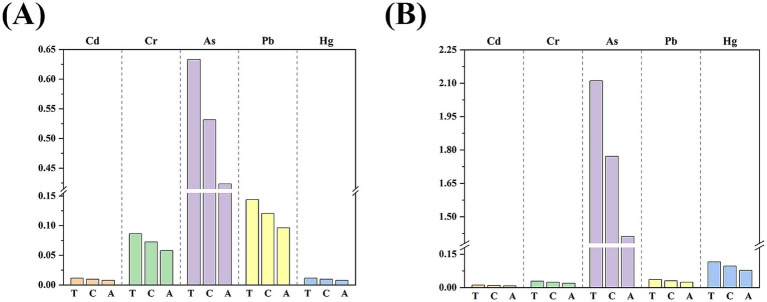
**(A)** Estimated daily intakes (median) of trace elements via rice consumption (μg/kg bw/day); **(B)** Hazard quotients of trace element exposure through ingestion of rice. A, adults; C, children; T, toddlers.

Figure 4A illustrated the median EDIs of heavy metals through rice consumption among different age groups, and the specific values were presented in Supplementary Table S5. The figure revealed that the daily intake of heavy metals, in descending order, were As, Pb, Cr, Cd, and Hg. The analysis indicated that adults exhibited the lowest EDIs of heavy metals from rice, with children showing moderate levels, and toddlers encountering the highest intake. This trend was consistent with the general observation that consumption of staple foods per unit of body weight tends to decline with the progression of age.

#### 3.5.2 Potential health risk assessment

Figure 4B and Supplementary Table S6 showed the Health Risk Index (HQ) values for five heavy metal elements (Cd, Cr, Pb, Hg, and As) ingested through rice consumption. Similar to the EDI, the HQ values for heavy metals were lowest in adults, intermediate in children and highest in toddlers. The data showed that for all age groups, the HQ values for Cd, Cr, Pb and Hg in rice were less than 1, suggesting that the potential health risk of these elements ingested through rice consumption is low. However, the HQ values for As were more than 1 for all age groups, indicating a potential health risk, especially in children and toddlers, where the HQ values for As were much greater than 1, suggesting that they have a relatively high of exposure to As and need special attention. In conclusion, with the exception of As, heavy metal elements ingested through rice consumption pose a low risk to health. Therefore, there is a need to further study the sources of As and to take effective measures to reduce the exposure risk of the rice consumption, especially toddlers and children.

## 4 Discussion

The present investigation detected the presence of Cr, As, Hg, Pb and Cd in a total of 6,632 rice samples gathered from Henan Province between 2020 and 2022. It was found that As had the highest detection rate, which was similar to the results of As detection rate (100%) in rice sampled from Jiangsu Province (21).

The content of heavy metals in rice in Henan Province was lower than in southern provinces, a phenomenon largely influenced by its geographic location, industrial structure and geological background. As an agricultural province in central China, Henan Province had relatively few industrial activities, especially compared to heavy industrial areas, which reduced the possibility of heavy metal pollution. Several studies had been conducted to show that heavy metal pollution was mainly concentrated in the south-central, southwestern and eastern coastal areas of China, where industrial activities were frequent (22–24). Rice had a certain capacity to absorb heavy metals, especially in heavy metal polluted areas, where the heavy metal content in rice might be higher. However, in Henan Province, where industrial activities were not as active as other provinces, the detection of heavy metals in rice was relatively low. When compared with the data reported from India, although the levels of cadmium and arsenic were slightly higher in Henan Province, but still below the maximum allowed strength (FMAC) for As (0.2 mg/kg) and Cd (0.4 mg/kg) in food as specified by the FAO/CODEX norm.

Through stratified analysis, it was observed that the detection rate of Cd in rice samples from rural areas was lower than that from urban areas. This was mainly affected by urban industrial emissions and transportation logistics. Specifically, Cd pollution was largely influenced by specific industrial activities, including metallurgical industry, chemical industry and electroplating industry. Wastewater, exhaust gases and solid wastes generated from these industrial areas were the main sources of Cd pollution in cities, which entered farmland through a variety of pathways resulting in the enrichment of heavy metals in rice and ultimately affecting the food chain (25–27). Cd emitted from automobile exhausts due to dense urban traffic also entered farmland through atmospheric deposition (28). In contrast, rural areas had less industrial activity, lower traffic flow and lower population density, which limited the sources and transmission pathways of Cd pollution. Scientific studies had also shown that urban soils contained higher levels of heavy metals than nearby suburban and rural soils, which, to some extent, contributed to the accumulation of heavy metals in rice (29).

Comparative analysis of the detection rates of heavy metals in rice in various regions of Henan Province revealed that the detection rate of Cr in rice in the eastern region was significantly higher than that in other regions. This difference is mainly attributed to the specific topography and farming practices in the eastern region. Specifically, the eastern region is dominated by plains, which are flat and vast, and have favorable conditions for agricultural cultivation. Pesticides and fertilizers are applied relatively frequently in the cultivated land in this region, and these chemicals often contain high levels of Cr, which leads to high detection rates of Cr in rice in this region (30). The high detection rate of Cd in rice in southern Henan Province was closely related to human activities such as industrial emissions, sewerage irrigation, and excessive use of Cd-containing fertilizers. Additionally, related studies indicated that Cd was more readily absorbed by plants in acidic soils or water sources, and the soils in the rainy regions of the south were generally acidic, which increased the probability of Cd exceeding the standard in food crops (31).

The health risk assessment indicated that the HQs value for As element in rice was greater than 1 in three age groups: adults, children, and toddlers. However, there were still some potential health risks associated with inorganic As exposure due to the consumption of rice in different age groups. In particular, toddlers and children were found to be at a higher health risk from rice consumption than adults. The emergence of this phenomenon was closely related to the growth and development stage of toddlers and children. At the stage, the functions of tissues and organs of toddlers were not yet fully mature, especially the relatively weak detoxification and excretion functions of metabolic organs such as the liver and kidneys. Coupled with a high intake of rice per unit of body weight, this made them more sensitive to heavy metals and other toxic and harmful substances. As they entered childhood, despite increased organ function, children still had a high intake of rice per unit of body weight compared to adults, so they were also at high risk, although the level of risk is slightly lower than that of toddlers (9, 32). Therefore, it is necessary to strengthen the monitoring of As to ensure that no rice with excessive As content enters the market and to protect the health and life safety of consumers.

Compared with other provinces or regions, relatively few researches have been conducted on heavy metal content in rice in Henan Province. The previous studies were primarily limited to some specific rice-growing areas and more polluted regions within Henan Province. To fill this gap, the study collected rice samples extensively from different regions and urban and rural areas in Henan Province, and accumulated a large amount of strong representative data. These data not only provide an important reference for quality and safety supervision of rice in Henan Province, but also enhance public awareness of the problem of heavy metal content in rice, and provide support for scientific research, policy making and agricultural environmental protection. However, there was limitation in this study in assessing the risk of heavy metals to human health. The study only focused on heavy metals ingested through rice and did not take into account heavy metals ingested through other foods such as meat, vegetables, and fruits in the daily diet. This could have led to an underestimation of the potential risk of heavy metals to human health. Therefore, future studies should provide a more comprehensive assessment of the contamination status of heavy metals and their risks to human health through multiple dietary and exposure routes in order to more accurately assess and protect public health.

In recent years, significant progress has been made in the technology of blocking and controlling heavy-metal pollution in rice, and thanks to the Action Plan for Soil Pollution Prevention and Control, implemented by the Chinese Government, the aggravating trend of heavy-metal pollution in arable soils has been effectively curbed. The Government must undertake sustained initiatives to alleviate heavy metal contamination in rice through the following. Firstly, reinforcing the regulation of industrial pollution sources, fostering the adoption of cleaner production technologies, and diminishing pollutant emissions. Secondly, extensively implementing soil remediation methodologies and selecting crop varieties with low heavy metal accumulation. Thirdly, augmenting food safety oversight, executing routine sampling inspections of rice, and establishing a robust traceability system. Lastly, elevating public health education and directing public to choose safe rice.

## 5 Conclusion

In this study, five heavy metals, As, Hg, Pb, Cr, and Cd were determined in rice from Henan Province by ICP-MS and health risk assessment was performed. Inter-regional differences indicated that the highest detection rates of the heavy metal Cd in rice were found in the southern part of Henan Province, Cr was detected at higher rates in the eastern Henan Province, and the level of contamination was generally higher in urban areas than in rural areas. The HQ value of As in rice exceeding recommended threshold, suggesting that exposure to As through rice consumption poses a potential risk to human health and should be emphasized by the relevant departments. In order to effectively mitigate the risk of heavy metal pollution, the relevant departments need to strengthen the supervision of industrial pollution, soil remediation, screening of low accumulation crops, and enhance food safety supervision, so as to reduce the risk of heavy metal pollution in rice in Henan Province, ensure the safety of agricultural products and public health. However, the study currently only assessed the risk of rice intake, and future studies should comprehensively assess heavy metal contamination and its risk to human health through dietary and exposure pathways.

## Data Availability

The raw data supporting the conclusions of this article will be made available by the authors, without undue reservation.
