# Supplementary material for: From farm to table: assessing the status and health risk assessment of heavy metal pollution in rice in Henan Province
Source: Front Public Health. 2025 Apr 17;13:1499653. doi: 10.3389/fpubh.2025.1499653 (PMC12043637; doi:10.3389/fpubh.2025.1499653)
Supplement: Supplementary file 1 [file Table_1.docx]

**SUPPLEMENTARY MATERIAL**

**Table S1 Microwave digestion reference conditions**

|  | Power (1200W)  change/% | Setting temperature/°C | Temperature rise time/min | Constant temperature time/min |
| --- | --- | --- | --- | --- |
| 1 | ０～８０ | 120 | 5 | 5 |
| 2 | ０～８０ | 150 | 5 | 10 |
| 3 | ０～８０ | 190 | 5 | 20 |

**Table S2 Daily consumption rates of staple foods and body weights for toddlers, children and adults.**

|  | IR of rice  (g day^-1^) | IR of wheat flour (g day^-1^) | BW (kg) | References |
| --- | --- | --- | --- | --- |
| Toddlers (2-5 years old) | 95.0^*^ | 55.9^*^ | 16.5 | (CAMEP, 2013a) |
| Children (6-17 years old) | 200.0^*^ | 117.6^*^ | 41.4 | (CAMEP, 2013b) |
| Adults （>18 years old) | 238.3 | 140.2 | 61.9 | (CAMEP, 2013c) |

**Table S3** Comparison of detection rates of various trace elements between rural and city.

| Heavy metals | Sample | | Detection rate (%) | | *P* |
| --- | --- | --- | --- | --- | --- |
|  | Urban | Rural | Urban | Rural |  |
| Cd | 1453 | 869 | 30.42 | 23.13 | <0.001 |
| Cr | 253 | 261 | 21.74 | 23.37 | 0.658 |
| Pb | 1446 | 866 | 2.49 | 1.85 | 0.314 |
| As | 507 | 463 | 99.21 | 100 | 0.157 |
| Hg | 253 | 261 | 1.58 | 2.30 | 0.787 |

**Table S4** Comparison of the detection rates of various trace elements in rice samples in different regions of Henan Province.

| Heavy  metals | Samples (Detection rate (%)) | | | | | *P* |
| --- | --- | --- | --- | --- | --- | --- |
|  | Eastern Henan | Western Henan | Southern Henan | Northern Henan | Central region of Henan |  |
| Cd | 425（30.2） | 166（20.5） | 555（**37.2**） | 378（21.5） | 798（24.1） | **<0.001** |
| Cr | 98（**32.7**） | 46（21.7） | 168（17.3） | 146（23.4） | 56（12.5） | **0.021** |
| Pb | 424（2.4） | 166（1.2） | 549（1.8） | 378（3.8） | 795（1.9） | 0.282 |
| As | 194（100） | 113（99.1） | 262（100） | 231（99.1） | 170（100） | 0.148 |
| Hg | 98（0） | 46（2.2） | 168（1.8） | 146（2.8） | 56（0） | 0.402 |

**Table S5 Estimated daily intakes (median) of trace elements via rice consumption. (μg kg-1 bw day-1)**

|  | Cd | Cr | Pb | Hg | As |
| --- | --- | --- | --- | --- | --- |
| Toddlers | 0.0115 | 0.0864 | 0.1439 | 0.0115 | 0.6333 |
| Children | 0.0097 | 0.0725 | 0.1208 | 0.0097 | 0.5314 |
| Adults | 0.0077 | 0.0577 | 0.0962 | 0.0077 | 0.4235 |

**Table S6 Hazard quotients of trace element exposure through ingestion of rice.**

|  | Cd | Cr | Pb | Hg | As |
| --- | --- | --- | --- | --- | --- |
| Toddlers | 0.0115 | 0.0288 | 0.0360 | 0.1152 | 2.1111 |
| Children | 0.0097 | 0.0242 | 0.0302 | 0.0966 | 1.7713 |
| Adults | 0.0077 | 0.0192 | 0.0241 | 0.0770 | 1.4116 |
